# Nomogram for cirrhosis in patients with chronic hepatitis B: A simple self-assessed scale for individual risk of cirrhosis

**DOI:** 10.1038/s41598-017-17685-4

**Published:** 2017-12-13

**Authors:** Zhiqiao Zhang, Jing Li, Peng Wang, Tingshan He, Yanling Ouyang, Yiyan Huang

**Affiliations:** Department of Infectious Diseases, Shunde Hospital of Southern Medical University, Shunde, Guangdong, China

## Abstract

The aim of this retrospective study was to establish a simple self-assessed scale for individual risk of cirrhosis in patients with chronic hepatitis B. A total of 1808 consecutive patients were enrolled and analyzed. According to the results of multivariate logistic regression analysis, a simple nomogram was calculated for cirrhosis. The area under receiver operating characteristic curves (AUROCs) were calculated to compare the diagnostic accuracy of nomogram with aspartate aminotransferase to platelet ratio index (APRI), fibrosis index based on the four factors (FIB-4), and S index. The AUROCs of nomogram for cirrhosis were 0.807 (adjusted AUROC 0.876) in model group and 0.794 (adjusted AUROC0.866) in validation group. DeLong’s test and Brier Score further demonstrated that nomogram was superior to APRI, FIB-4 and S index in both model group and validation group. The patients with nomogram <0.07 could be defined as low risk group with cirrhosis prevalence lower than 4.3% (17/397). The patients with nomogram >0.52 could be defined as high risk group with cirrhosis prevalence higher than 73.0% (119/163). In conclusion, as a self-assessed style, simple, non-invasive, economical, convenient, and repeatable scale, nomogram is suitable to serve as a massive health screening tool for cirrhosis in CHB patients and further external validation is needed.

## Introduction

As a public health problem, hepatitis B virus (HBV) affected 350 million people in the world. The corresponding 5-year cumulative incidences of cirrhosis were 8% and 17% in hepatitis B e antigen (HBeAg) positive patients in East Asian countries and European countries, whereas it were 13% and 38% in HBeAg negative patients, respectively^1^. For patient with cirrhosis, the 5-year cumulative incidences of hepatocellular carcinoma (HCC) were 17% in East Asia and 10% in the Western Europe and the United States^1^. For patients with compensated cirrhosis, the 5-year cumulative incidence of liver decompensation was 15% in European and Asian studies^2–4^. The 5-year liver related death incidences in patients with compensated cirrhosis were 15% in Europe and 14% in East Asia, whereas it was 70% to 85% for patients with uncompensated cirrhosis^4–6^. There were over 200,000 and 300,000 chronic HBV carriers died each year from cirrhosis and HCC, respectively^7,8^. Therefore, the early detection of cirrhosis is of significance for prevention of HCC and cirrhosis.

Liver biopsy is the best available standard in assessing cirrhosis but is limited by its invasiveness and sampling error^9,10^. Transient elastography (TE) has a better diagnostic value in detecting hepatic fibrosis. However, it is difficult to obtain measurement data in case of obesity, ascites and limited operator experience^11^. It has been found that acute hepatitis, extrahepatic cholestasis and congestion would result in elevated false positive and reduce the diagnostic accuracy^12,13^. In addition, TE is not readily available in most primary hospitals in developing countries. From the perspective of clinical practice and cost-effectiveness, an ideal screening tool for cirrhosis should be a simple, non-invasive, economical, convenient and repeatable method. Furthermore, personalized risk assessment of cirrhosis represents a challenge for management of patient with chronic hepatitis B.

Nomogram which derived from hazard functions has been applied to various diseases as a straightforward predictive tool^14,15^. The nomogram is convenient for clinicians and patients to assess the probability of disease without complex formula. In addition, nomogram can provide straightforward individual risk assessment, which is readily comprehensible for patients without medical knowledge. Therefore, nomogram improves the clinical significance from group-level to individual-level and is favorable for clinicians and patients. The aim of this study was to build and validate a simple nomogram for assessment of cirrhosis in patients with chronic hepatitis B (CHB).

## Patients and Methods

### Patients

This retrospective study included eligible patients diagnosed as chronic hepatitis B and had undergone liver biopsy in department of infectious diseases of Shunde Hospital of Southern Medical University, between January 2008 and November 2014. The Patients were enrolled based on the following criteria: chronic hepatitis B was defined as hepatitis B surface antigen (HBsAg) positivity for more than 6 months. The exclusion criteria were as follows: liver cancer; co-infection with hepatitis C virus, hepatitis D virus or human immunodeficiency virus; autoimmune liver diseases such as autoimmune hepatitis, primary biliary cirrhosis, and primary sclerosing cholangitis; hereditary and metabolic liver diseases such as Wilson’s disease, hemochromatosis, and α−1-antitrypsin deficiency. Therefore, there were 344 patients excluded from the current study according to above criteria. There were no significant differences in terms of demographic and clinical parameters between patients included and excluded (data not shown).

All data collections and clinical investigations were performed according to the principles of Declaration of Helsinki. The study was approved by the ethics committee of Shunde Hospital of Southern Medical University. We performed this study according to the STARD recommendations for the optimal quality in reporting diagnostic accuracy.

### Liver biopsy

Liver biopsies were performed by two experienced physicians using a 16-gauge needle (16 G biopsy Menghini’s needle, ShangHai). Only the liver tissues with a length more than 1.5 cm were recruited in the present study. The specimens were fixed, paraffin-embedded and stained with haematoxylin and eosin (HE). Histological grading of necro-inflammation (G0–G4) and staging of the liver fibrosis (S0–S4) were carried out according to Scheuer classification^16^ by one experienced pathologist blinded to the clinical data. In the study, cirrhosis was defined as fibrosis stage = S4.

### Serum markers and noninvasive models

All patients systematically underwent complete biochemical workups, ultrasonography and liver biopsy within 2 days. Blood samples of the subjects were obtained before liver biopsy. Biochemical tests were performed in laboratory of Shunde Hospital of Southern Medical University for alanine aminotransferase (ALT,U/L), aspartate aminotransferase (AST,U/L), γ-glutamyl transferase (GGT, U/L), total bilirubin (TBIL, mmol/L), white blood cell (WBC, 10^9/L), hemoglobin (HGB, g/L), platelet (PLT, 10^9/L), α-fetoprotein (AFP, ng/ml), hyaluronic acid (HA, μg/L), fasting plasma glucose (FPG, mmol/L), total cholesterol (TC, mmol/L), triglycerides (TG, mmol/L), high-density lipoprotein cholesterol (HDL, mmol/L); low-density lipoprotein cholesterol (LDL, mmol/L). The serum HBV-DNA level was detected with a Real-Time polymerase chain reaction (PCR) System (ABI7700; Applied Shenzhen city Daeran Biological Engineering Co Ltd, Shenzhen, Guangdong, CHN). HBsAg was measured with CLIA systems (Abbott ARCHITECT i2000 SR system, Abbott Laboratories, Abbott Park, IL, USA).

The formulas of aspartate aminotransferase to platelet ratio index (APRI), fibrosis index based on the four factors (FIB-4), and S index were calculated as described in the original articles^17–19^. APRI: (AST/[ULN]/PLT [109/L])*100; FIB-4: (age [year]*AST [U/L])/{ (PLT [109/L])* (ALT [U/L])^1/2^}; S index: 1000*GGT/ (PLT*ALB^2^).

### Standardisation of AUROC according to the prevalence of fibrosis stages

It has been found that the prevalence of different liver fibrosis stages may be a major factor of variability in assessing the diagnostic accuracy of noninvasive model. Therefore, AUROC should be adjusted according to the prevalence of fibrosis stages using the difference between advanced and non-advanced fibrosis (DANA) method^20^. DANA was calculated according to the following formula: DANA = [(prevalence F4*4)/ (prevalence F4)] – [prevalence F1 + prevalence F2*2 + prevalence F3*3/ (prevalence F0 + prevalence F1 + prevalence F2 + prevalence F3)]. The adjusted AUROC (AdjAUROC) was calculated as follow: AdjAUROC = observed AUROC + 0.1056* (2.5 –DANA).

### Data Availability

The datasets analyzed during the current study are available from the corresponding author on reasonable request.

### Statistical analysis

Continuous data were expressed as mean ± standard deviation or median (minimum, maximum) depending on the normality of variables. Continuous variables were compared by t-test or Mann-Whitney U test as appropriate. Categorical variables were compared by chi-squared test or Fisher’s exact test as appropriate. All variables that significantly associated with fibrosis in univariate logistic regression analysis were included in forward stepwise multivariate logistic regression analysis to derive a nomogram for cirrhosis. The area under receiver operating characteristic curves (AUROCs) were calculated to evaluate the diagnostic accuracy of nomogram in predicting cirrhosis and compared by DeLong’s test^21^.

Statistical analyses were performed using SPSS 19.0 (SPSS Inc., Chicago, IL). All statistical tests were two-sided. *P* < 0.05 was considered statistically significant.

## Results

### The characteristics of subjects in model group and validation group

A total of 1808 patients were recruited into the present study with a mean age of 33.3 ± 9.6 years. Of all patients in the current study, 1422 (78.7%) were male and 386 (21.3%) were female, 1143 (63.2%) were HBeAg positive and 665 (36.8%) were HBeAg negative. The fibrosis stages were 275 (15.2%) in S1, 656 (36.3%) in S2, 495 (27.4%) in S3 and 382 (21.1%) in S4. The inflammation grades were 113 (6.3%) in G1, 815 (45.1%) in G2, 643 (35.6%) in G3 and 237 (13.1%) in G4.

The patients were randomly divided into model group (n = 1080) and validation group (n = 728) using whole group random sampling method using SPSS 19.0. The baseline characteristics of patients in model group and validation group were summarized in Table 1.

**Table 1:** Characteristics of patients in model group and validation group.

	Model group	Validation group	Test value	*P*
	n = 1080	n = 728		
Age (year)	33.1 ± 9.5	33.5 ± 9.7	−0.893	0.372
Male (n, %)	854 (79.1)	568 (78.0)	0.29	0.593
ALT (U/L)	74 (4,398)	58 (2,390)	−3.074	0.002
AST (U/L)	53 (2,396)	51 (4,410)	−0.410	0.682
GGT (U/L)	45 (5,649)	74 (11,999)	−8.926	<0.001
Albumin (g/L)	44.2 ± 4.9	44.0 ± 4.8	1.835	0.067
Globulin (g/L)	27.9 ± 4.8	27.3 ± 4.6	2.336	0.020
TBIL (μmol/L)	15 (4.2,34)	16.7 (4,44)	−4.610	<0.001
Creatinine (μmol/L)	80.5 ± 24.8	78.5 ± 17.8	1.192	0.056
FPG (mmol/L)	4.7 ± 1.1	4.7 ± 1.1	−0.564	0.573
TC (mmol/L)	4.4 ± 1.0	4.4 ± 1.0	0.366	0.714
TG (mmol/L)	1.2 ± 0.6	1.2 ± 0.6	−1.735	0.083
HDL (mmol/L)	1.3 ± 0.4	1.3 ± 0.4	0.783	0.434
LDL (mmol/L)	2.3 ± 0.8	2.3 ± 0.7	0.825	0.409
WBC (10^9/L)	5.7 ± 1.6	5.7 ± 1.8	−0.218	0.828
HGB (g/L)	144.30 ± 17.0	142.9 ± 15.9	−0.470	0.638
PLT (10^9/L)	183.7 ± 54.6	177.6 ± 55.9	−2.295	0.022
HBV DNA positive (n, %)	839 (77.7)	559 (76.8)	0.2	0.654
HBeAg positive (n, %)	679 (62.9)	464 (63.7)	0.14	0.708
Anti-virus (n, %)	218 (20.2)	152 (20.9)	0.13	0.720
AFP (ng/ml)	5.1 (0.1,1222)	9.8 (2,9000)	−11.053	<0.001
HA (μg/L)	68 (1.4,947)	119 (5,960)	−12.566	<0.001
Inflammation Grade 1 (n, %)	68 (6.3)	45 (6.2)	2.5	0.479
Inflammation Grade 2 (n, %)	480 (44.4)	335 (40.0)		
Inflammation Grade 3 (n, %)	398 (36.9)	245 (33.7)		
Inflammation Grade 4 (n, %)	134 (12.4)	103 (14.1)		
Fibrosis Stage 1 (n, %)	166 (15.4)	109 (15.0)	1.54	0.674
Fibrosis Stage 2 (n, %)	402 (37.2)	254 (35.0)		
Fibrosis Stage 3 (n, %)	286 (26.5)	209 (28.7)		
Fibrosis Stage 4 (n, %)	226 (20.9)	156 (21.4)		

Note: Continuous variables were expressed as mean ± standard deviation or median (minimum, maximum) as appropriate; ALT, alanine aminotransferase; AST, aspartate aminotransferase; GGT, γ-glutamyl transferase; TBIL, total bilirubin; FPG, fasting plasma glucose; TC, total cholesterol; TG, triglyceride; HDL, high-density lipoprotein cholesterol; LDL, low-density lipoprotein cholesterol; WBC, white blood cell; HGB, hemoglobin; PLT, platelet; AFP, α-fetoprotein; HA, hyaluronic acid.

### Nomogram for cirrhosis

**Figure 1:**
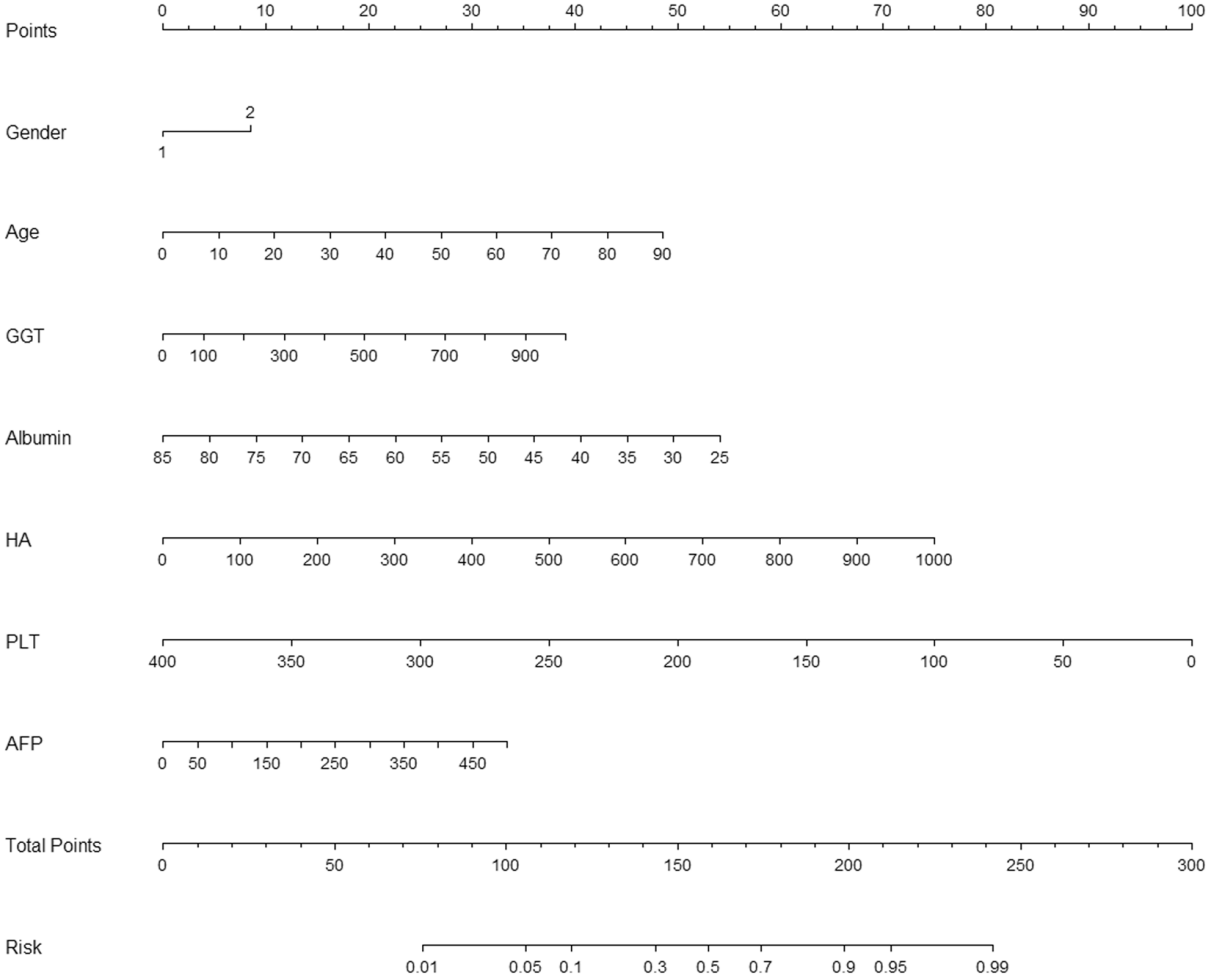
Nomogram for cirrhosis. AFP, α-fetoprotein; GGT, γ-glutamyl transferase; HA, hyaluronic acid; PLT, platelet.

All variables that significantly associated with cirrhosis in univariate logistic regression analysis were included in multivariate logistic regression analysis (forward stepwise method) to derive a nomogram for cirrhosis (Table 2 and Fig. 1). At last, age, gender, α-fetoprotein (AFP), γ-glutamyl transferase (GGT), hyaluronic acid (HA), Albumin and platelet (PLT) were included in the nomogram for cirrhosis.

**Table 2:** Univariate and multivariate analysis for variables included in the nomogram for prediction of cirrhosis.

	S1–3	S4	*P*	Univariate regression		Multivariate regression		
	n = 854	n = 226		OR	*P*	coefficient	*P*	OR (95% CI)
Gender (n,%)	658 (80.2)	196 (86.7)	0.002	1.993 (1.448–2.742)	<0.001	0.484	0.010	1.622 (1.122,2.345)
Age (year)	32.0 ± 8.9	37.6 ± 10.5	<0.001	1.058 (1.046–1.070)	<0.001	0.030	<0.001	1.030 (0.107,1.044)
ALT (U/L)	74 (5,399)	59 (6,410)	0.047	0.998 (0.997–1.0)	0.066			
AST (U/L)	52 (2,390)	51 (11,420)	0.577	0.001 (0.999,1.002)	0.284			
GGT (U/L)	44 (96,631)	69 (11,900)	<0.001	1.004 (1.003–1.006)	<0.001	0.002	0.005	1.002 (1.001,1.003)
Albumin (g/L)	44.9 ± 4.8	42.6 ± 4.9	<0.001	0.892 (0.868–0.916)	<0.001	−0.053	<0.001	0.948 (0.920,0.977)
Globulin (g/L)	27.5 ± 4.5	29.3 ± 5.3	<0.001	1.079 (1.053–1.105)	<0.001			
TBIL (μmol/L)	15 (4.0,38.0)	17 (94.0,39.0)	<0.001	1.016 (1.007–1.024)	<0.001			
Creatinine (μmol/L)	79.8 ± 17.7	83.4 ± 42.0	0.204	1.008 (1.002–1.014)	0.010			
FPG (mmol/L)	4.6 ± 1.0	4.7 ± 1.5	0.413	1.077 (0.981–1.182)	0.119			
TC (mmol/L)	4.4 ± 1.0	4.3 ± 1.0	0.062	0.827 (0.736–0.929)	<0.001			
TG (mmol/L)	1.1 ± 0.6	1.2 ± 0.6	0.138	1.120 (0.947–1.324)	0.187			
HDL (mmol/L)	1.4 ± 0.4	1.2 ± 0.4	<0.001	0.472 (0.351–0.635)	<0.001			
LDL (mmol/L)	2.4 ± 0.8	2.2 ± 0.7	0.028	0.841 (0.720–0.982)	0.029			
WBC (10^9/L)	5.7 ± 1.5	5.4 ± 1.6	0.007	0.943 (0.878–1.013)	0.109			
HGB (g/L)	144.2 ± 16.9	139.8 ± 17.0	<0.001	0.985 (0.979–0.992)	<0.001			
PLT (10^9/L)	192.1 ± 52.3	151.9 ± 51.2	<0.001	0.984 (0.981–0.986)	<0.001	−0.014	<0.001	0.986 (0.984,0.989)
AFP (ng/ml)	5.2 (0.1,1222)	11 (0.9,1909)	<0.001	1.004 (1.002–1.005)	<0.001	0.002	0.024	1.002 (1.001,1.003)
HA (μg/L)	67 (4.7,800)	118 (5,900)	<0.001	1.006 (1.005–1.007)	<0.001	0.004	<0.001	1.004 (1.003,1.005)
Constant						1.117		

Note:ALT, alanine aminotransferase; AST, aspartate aminotransferase; GGT,γ-glutamyl transferase; TBIL, total bilirubin; FPG, fasting plasma glucose; TC, total cholesterol; TG, triglyceride; HDL, high-density lipoprotein cholesterol; LDL, low-density lipoprotein cholesterol; WBC, white blood cell; HGB, hemoglobin; PLT, platelet; AFP, α-fetoprotein; HA, hyaluronic acid; OR, odds ratio.

Nomogram = exp (1.117 + 0.03 × Age + 0.002 × GGT-0.053 × Albumin + 0.004 × HA-0.014 × PLT-0.002 × AFP + 0.484 × Gender)/{1 + exp (1.117 + 0.03 × Age + 0.002 × GGT-0.053 × Albumin + 0.004 × HA-0.014 × PLT-0.002 × AFP + 0.484 × Gender)}.

### Diagnostic accuracy of nomogram for cirrhosis in model group and validation group

**Figure 2:**
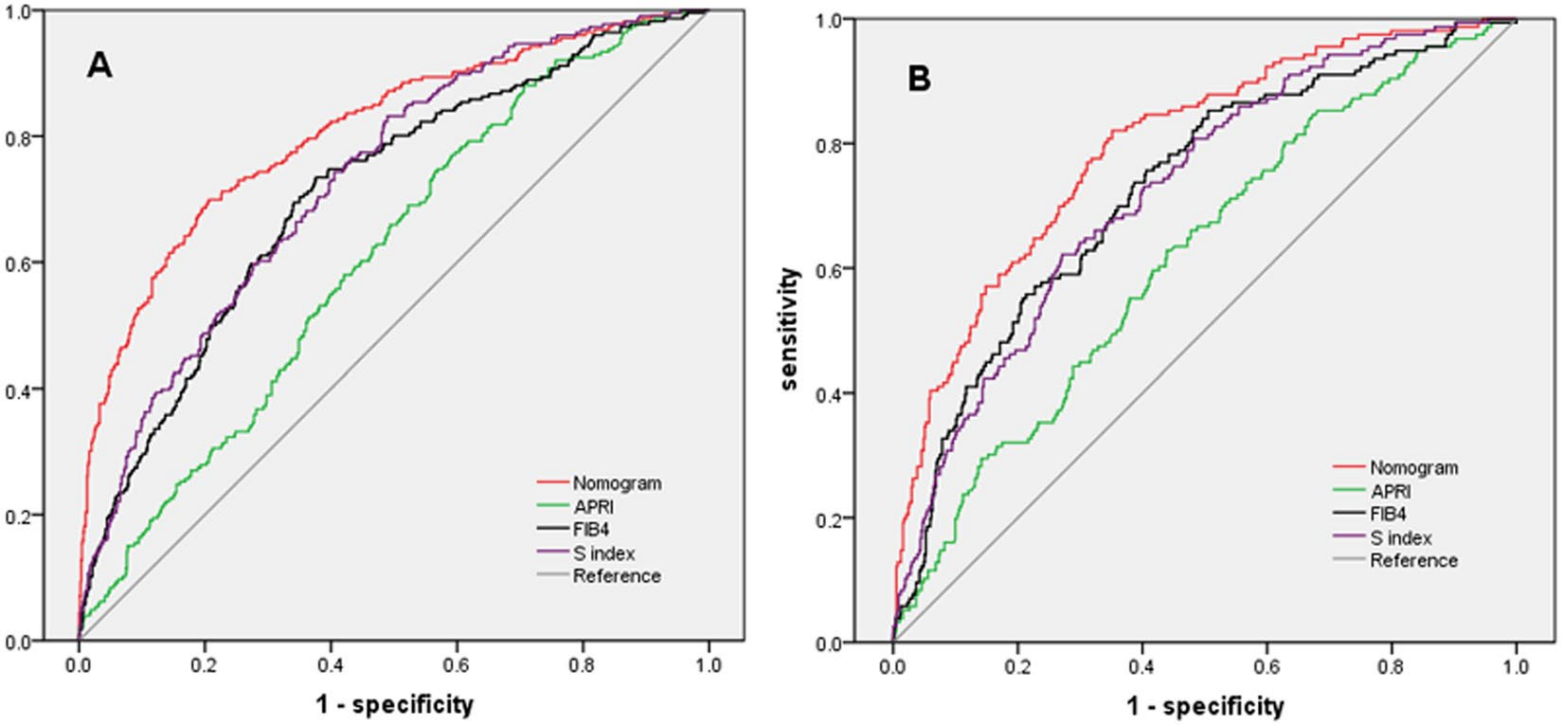
ROC curves of four predictive indexes for cirrhosis in different groups: (**A**) model group; (**B**) validation group.

The receiver operating characteristic curve of nomogram was drawn to assess the diagnostic accuracy for cirrhosis (Fig. 2). The AUROCs of nomogram, APRI, FIB-4 and S index for cirrhosis were 0.807 (AdjAUROC 0.876, 95%CI 0.773–0.841), 0.609 (AdjAUROC0.678, 95%CI 0.570–0.648), 0.710 (AdjAUROC0.779, 95%CI 0.673–0.748), and 0.730 (AdjAUROC0.799, 95%CI 0.695–0.766) in model group. In validation group, the AUROCs of nomogram, APRI,FIB-4 and S index were 0.794 (AdjAUROC0.866, 95%CI 0.755–0.834), 0.618 (AdjAUROC0.690, 95%CI 0.569–0.666), 0.727 (AdjAUROC0.796, 95%CI 0.682–0.771), and 0.726 (AdjAUROC0.794, 95%CI 0.680–0.773).Comparisons of AUROCs using DeLong’s test method demonstrated that nomogram was significantly superior to APRI, FIB-4 and S index for both model group and validation group (all *P* < 0.001).

#### Calibration curve of nomogram for cirrhosis

**Figure 3:**
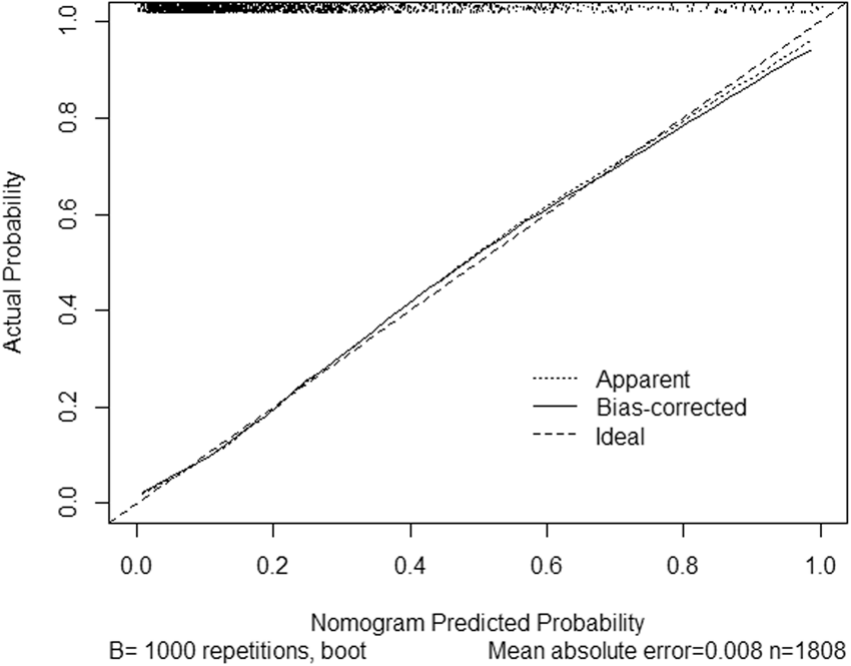
Internal calibration curve of nomogram for cirrhosis. Calibration curve showed good agreement between the predicted and observed probabilities for cirrhosis.

The further calibration curve was showed in Fig. 3. A calibration plot compares the model’s predicted probabilities and observed proportions. The diagonal line reflects the ideal situation (predicted probability = observed proportion). The calibration curve (Fig. 3) showed that the nomogram model appeared to be well-calibrated and there was a good agreement between the observed and predicted probabilities of cirrhosis.

### The Brier Score of four diagnostic indexes

The Brier Score is the mean squared error of the probability forecasts over the verification sample, ranging from o to 1. Brier Score is a proper score function to measure the accuracy of probabilistic predictions and widely used for the verification of probability forecasts^22,23^. Therefore, the closer the Brier Score is to 0, the better the calibration of the model.

The Brier Score of nomogram, APRI, FIB-4 and S index for cirrhosis were 0.1217, 0.1627, 0.1523, and 0.1505 in model group. The Brier Score of nomogram, APRI, FIB-4 and S index were 0.1334, 0.1649, 0.1560, and 0.1537 in validation group. The Brier Score of nomogram was significantly less than that of other three indexes, indicating that nomogram had the highest predictive accuracy in four diagnostic indexes.

### Clinical utility of nomogram for cirrhosis

The optimal cut-off values for predicting fibrosis were determined according to positive likelihood ratio (PLR) nearly 10.0 for high risk group and negative likelihood ratio (NLR) nearly 0.1 for low risk group^24^.

For cirrhosis, the high risk cut-off value of 0.52 showed a PLR 10.01, a specificity 96.9%, and a negative predictive value 84.0%. The low risk cut-off value of 0.07 showed a NLR 0.17, a sensitivity 95.6%, and a positive predictive value 25.9%. The low positive predictive value (25.9%) for low risk cut-off value 0.07 was associated with low cirrhosis prevalence (21.1%) in the present study.

The patients with nomogram < 0.07 could be defined as low risk group with cirrhosis prevalence lower than 4.3% (17/397). The cirrhosis prevalence of patients in middle risk group (0.07 ≤ nomogram index ≤ 0.52) was 19.7% (246/1248). The patients with nomogram > 0.52 could be defined as high risk group with cirrhosis prevalence higher than 73.0% (119/163).

### Diagnostic accuracy of nomogram for patients without antivirus therapy

**Figure 4:**
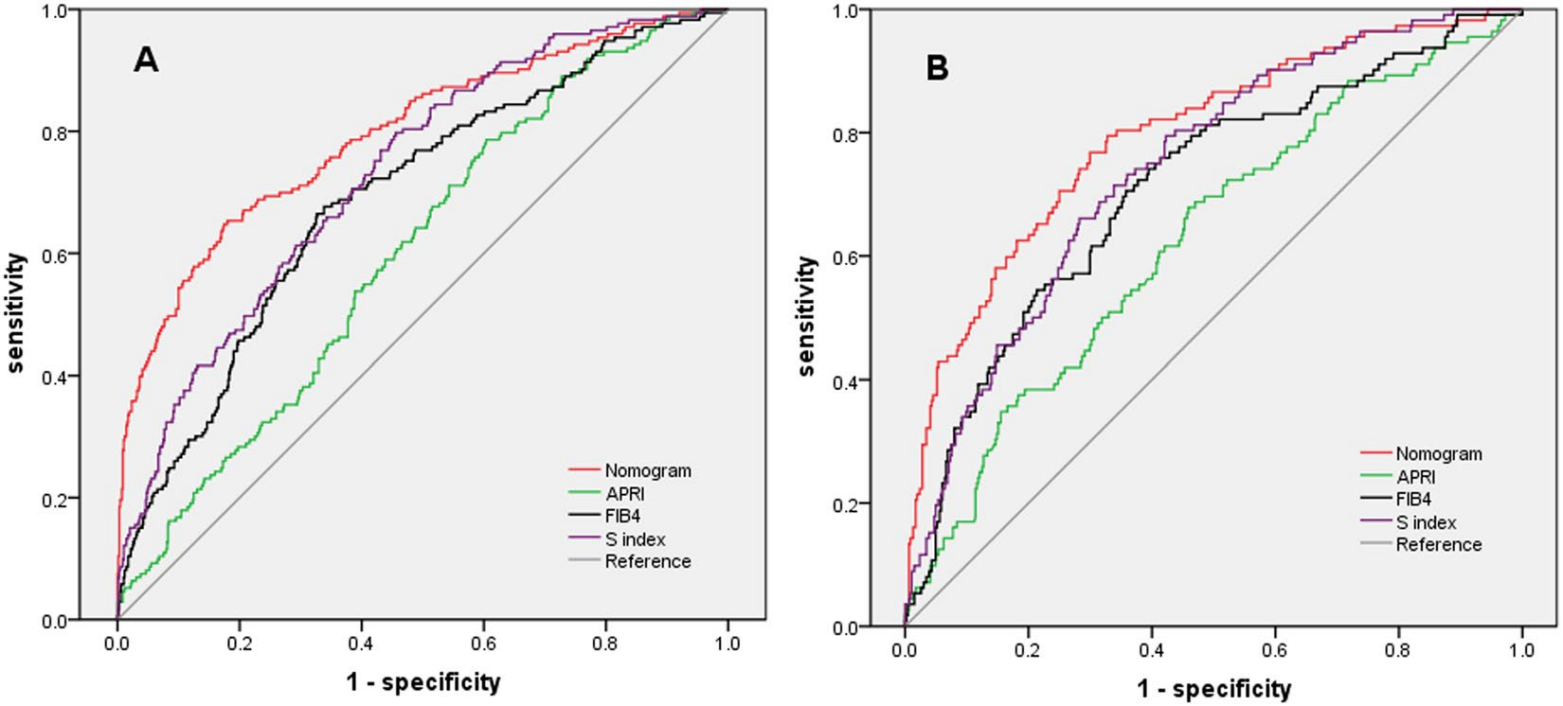
ROC curves of four predictive indexes for cirrhosis in patients without antivirus therapy: (**A**) model group; (**B**) validation group.

We further explored the diagnostic accuracy of nomogram for patients without antivirus therapy (Fig. 4). For patients without antivirus therapy in model group (n = 862), the AUROCs of nomogram, APRI, FIB-4 and S index for cirrhosis were 0.795 (95%CI 0.754–0.835), 0.603 (95%CI 0.558–0.647), 0.692 (95%CI 0.648–0.736), and 0.731 (95%CI 0.691–0.772). For patients without antivirus therapy in validation group (n = 576), the AUROCs of nomogram, APRI,FIB-4 and S index were 0.794 (95%CI 0.726–0.841), 0.628 (95%CI 0.570–0.685), 0.711 (95%CI 0.657–0.765), and 0.742 (95%CI 0.694–0.790). Comparisons of AUROCs using DeLong’s test method showed that nomogram was significantly superior to APRI,FIB-4 and S index in patients without antivirus therapy.

## Discussion

A nomogram was derived for detection of cirrhosis in CHB patients. The AUROCs of nomogram for cirrhosis were 0.807 (AdjAUROC 0.876) in model group and 0.794 (AdjAUROC0.866) in validation group. DeLong’s test and Brier Score demonstrated that nomogram was superior to other three indexes in both model group and validation group for fibrosis. The patients with nomogram < 0.07 could be defined as low risk group with cirrhosis prevalence lower than 4.3% (17/397). The patients with nomogram > 0.52 could be defined as high risk group with cirrhosis prevalence higher than 73.0% (119/163).

Nomogram for cirrhosis involved PLT, age, AFP, GGT, HA, Albumin, and gender. All these parameters had been found to be correlated with advanced fibrosis in previous studies. Platelet count was related with portal hypertension and advanced fibrosis^25^. Age had been applied as a surrogate marker of disease duration and was correlated with advanced fibrosis^25^. AFP had been found to be correlated with hepatic impair and chronic fibrosis, thus AFP was helpful to differential diagnosis of fibrosis stage^26,27^. Bile duct lesions caused by HBV infection could partially explain the elevated GGT and patients with elevated GGT often had significantly higher fibrosis scores^28,29^. It had been found that serum HA level increased in chronic liver diseases and elevated serum HA was helpful to identify the progressive liver damage^30,31^. The albumin was exclusively synthesized in live and albumin level fell along with the decline of hepatic synthetic function in patients with worsening liver fibrosis^32^. Albumin level decreased in case of cirrhosis and had been utilized in Child-Pugh classification^33^. Gender had been utilized as a predictor for advanced fibrosis and cirrhosis in the predictive index suggested by Wang *et al*.^34^. In the current study, these above parameters were confirmed as independent influence factors in multivariate logistic regression analysis.

The diagnostic accuracy of APRI, FIB-4 and S index in the current study was different to that in previous studies^17–19^. The differences of APRI, FIB-4 and S index in predicting cirrhosis might be related to the following reasons. First, FIB-4 was constructed in patients with human immunodeficiency virus (HIV)/hepatitis C virus (HCV) co-infection, whereas APRI was derived from patients with HCV. HBV, HCV and HIV infection have different influences on clinical characteristics, progression of fibrosis and diagnostic markers. Second, the influence of different prevalence of fibrosis stages in various studies should be taken into account for assessment of diagnostic accuracy of noninvasive indexes. Third, the inclusion of GGT, HA, albumin, age, and gender might enhance the efficiency of nomogram in predicting cirrhosis compared with APRI, FIB-4 and S index.

This nomogram is a good choice for massive screening in detecting cirrhosis as an alternative to liver biopsy or examinations for the following reasons. First, this nomogram is easy to calculate by patients themselves without complex mathematical calculation. Therefore, this nomogram provides a self-assessed scale of individual risk of cirrhosis to patients themselves. Second, this nomogram score is directly translated to a relative individualized risk probability of cirrhosis, which is easy to understand for patients without medical knowledge. Third, all relevant parameters of this nomogram are readily available in routine health examinations with no additional cost. Fourth, this nomogram is easily applicable for clinical practice because this nomogram does not need additional equipments, which is of importance for most primary hospitals in developing countries. Fifth, the patients with nomogram <0.07 could be defined as low risk of cirrhosis with a correct rate of 95.7%. In summary, as a self-assessed style, simple, non-invasive, economical, convenient and repeatable scale, it is worth considering utilizing this nomogram as a massive screening tool in selecting patients for further imaging examinations or liver biopsy.

The present study has several strengths as follows. Firstly, the present study finally included 1808 patients with CHB, providing a convincing conclusion for diagnostic accuracy of cirrhosis. Secondly, the AUROCs in the current study were adjusted using DANA method to adjust the influence of different prevalence of fibrosis stages, providing standard results for further comparisons in different studies. Thirdly, the Brier Score of four indexes further demonstrated that nomogram has the highest predictive accuracy in four diagnostic indexes.

The present study has three limitations which should be taken into account. First, this nomogram did not include some valuable variables such asα2-macroglobulin and body mass index due to the present study was a retrospective study. Second, the present study was a single center study, which might reduce the representative of the study population. Large scale and multi-center studies are needed to externally validate the diagnostic accuracy of nomogram. Third, HA is not a common parameter in conventional health examination and may be a limitation for the application of nomogram in different study population. Therefore, this proposed nomogram requires further external studies and confirmations.

In conclusion, as a self-assessed style, simple, non-invasive, economical, convenient, and repeatable scale, nomogram is suitable to serve as a massive health screening tool for cirrhosis in CHB patients and further external validation is needed.
